# Spectral-based thickness profiling of the corpus callosum enhances anomaly detection in fetal alcohol spectrum disorders

**DOI:** 10.3389/fnins.2023.1289013

**Published:** 2023-11-06

**Authors:** Justine Fraize, Yann Leprince, Monique Elmaleh-Bergès, Eliot Kerdreux, Richard Delorme, Lucie Hertz-Pannier, Julien Lefèvre, David Germanaud

**Affiliations:** ^1^UNIACT, NeuroSpin, Frederic Joliot Institute, Centre d’études de Saclay, CEA Paris-Saclay, Gif-sur-Yvette, France; ^2^InDEV, NeuroDiderot, Inserm, Université Paris Cité, Paris, France; ^3^Department of Pediatric Radiologic, Robert-Debré Hospital, AP-HP, Centre of Excellence InovAND, Paris, France; ^4^Department of Child and Adolescent Psychiatry, Robert-Debré Hospital, AP-HP, Centre of Excellence InovAND, Paris, France; ^5^Institut de Neurosciences de La Timone, CNRS, Aix-Marseille Université, Marseille, France; ^6^Department of Genetics, Robert-Debré Hospital, AP-HP, Centre de Référence Déficiences Intellectuelles de Causes Rares, Centre of Excellence InovAND, Paris, France

**Keywords:** corpus callosum, fetal alcohol spectrum disorders, fetal alcohol syndrome, spectral analysis, diagnostic imaging, scaling analysis, microcephaly

## Abstract

**Introduction:**

Fetal alcohol spectrum disorders (FASD) range from fetal alcohol syndrome (FAS) to non-syndromic forms (NS-FASD). The neuroanatomical consequences of prenatal alcohol exposure are mainly the reduction in brain size, but also focal abnormalities such as those of the corpus callosum (CC). We previously showed a narrowing of the CC for brain size, using manual measurement and its usefulness to improve diagnostic certainty. Our aim was to automate these measurements of the CC and identify more recurrent abnormalities in FAS subjects, independently of brain size reduction.

**Methods:**

We developed a fast, automated, and normalization-free method based on spectral analysis to generate thicknesses of the CC continuously and at singular points (genu, body, isthmus, and splenium), and its length (LCC). We applied it on midsagittal section of the CC extracted from T1-anatomical brain MRI of 89 subjects with FASD (52 FAS, 37 NS-FASD) and 126 with typically development (6–20 y-o). After adjusting for batch effect, we compared the mean profiles and thicknesses of the singular points across the 3 groups. For each parameter, we established variations with age (growth charts) and brain size in the control group (scaling charts), then identified participants with abnormal measurements (<10th percentile).

**Results:**

We confirmed the slimming of the posterior half of the CC in both FASD groups, and of the genu section in the FAS group, compared to the control group. We found a significant group effect for the LCC, genu, median body, isthmus, and splenium thicknesses (*p* < 0.05). We described a body hump whose morphology did not differ between groups. According to the growth charts, there was an excess of FASD subjects with abnormal LCC and isthmus, and of FAS subjects with abnormal genu and splenium. According to the scaling charts, this excess remained only for LCC, isthmus and splenium, undersized for brain size.

**Conclusion:**

We characterized size-independent anomalies of the posterior part of the CC in FASD, with an automated method, confirming and extending our previous study. Our new tool brings the use of a neuroanatomical criterion including CC damage closer to clinical practice. Our results suggest that an FAS signature identified in NS-FASD, could improve diagnosis specificity.

## 1. Introduction

The diagnosis of fetal alcohol spectrum disorders (FASD) encompasses the pathological consequences of prenatal alcohol exposure, which range from fetal alcohol syndrome (FAS) to non-syndromic, non-specific forms (NS-FASD) (Astley, 2004; Cook et al., 2016; Hoyme et al., 2016). The diagnosis of fetal alcohol syndrome (FAS) is determined by a consensual set of clinical features, which include facial dysmorphia, growth retardation, and microcephaly. On the other hand, the diagnosis of non-syndromic fetal alcohol spectrum disorder (NS-FASD), which associate neurodevelopmental disorders with prenatal alcohol exposure, remains probabilistic. The main target of the teratogenic effects of ethanol is the brain, leading individuals with FASD to exhibit not only a smaller brain but also recurrent focal brain abnormalities that are detectable via magnetic resonance imaging (MRI).

One of the most commonly reported focal brain abnormalities is the corpus callosum. This large bundle of white matter fibers composed of interhemispheric homotopic axonal projections turns out to be a privileged indicator of the consequences of prenatal alcohol exposure. The last four large radiological description studies reported thinning, hypoplasia, complete or partial agenesis of the corpus callosum in series composed of children either prenatally exposed or with a diagnosis of FASD (Autti-Rämö et al., 2002; Astley, 2004; Boronat et al., 2017; Treit et al., 2020; Fraize et al., 2023c). The prevalence of corpus callosum anomalies that are visible to the naked eye, although partly subjective, could approach 3 to 10% in the FASD population. Computational neuroimaging has sought to uncover more subtle anomalies or more objective morphometric descriptions. Several studies conducted on subjects with FASD have suggested that the midsagittal corpus callosum area (Riley et al., 1995; Sowell et al., 2001; Astley et al., 2009; Dodge et al., 2009; Fraize et al., 2023c), thickness (Yang et al., 2012a), and volume (Gautam et al., 2014; Biffen et al., 2020; Inkelis et al., 2020) may be reduced compared to controls. These changes have also been correlated with the amount of prenatal alcohol consumption (Biffen et al., 2017; Jacobson et al., 2017). In addition to a global reduction in the size of the corpus callosum in FASD, the posterior region appears to be more severely affected in size (Sowell et al., 2001; Dodge et al., 2009; Fraize et al., 2023a) or in shape, resulting in a flattened or misshapen appearance (Sowell et al., 2001; Bookstein et al., 2002). However, these results need to be qualified, because even within the same team, they were not always confirmed. In the study by Yang et al., where three series from different sites were pooled, the thinning on the posterior part initially described (Sowell et al., 2001) was found to be significant in only one sub-group (Yang et al., 2012a). Despite these detailed descriptions, no neuroanatomical criterion referring explicitly to specific anomalies of the corpus callosum have been added to the diagnostic guidelines (Astley, 2004; Cook et al., 2016; Hoyme et al., 2016). Yet, we recently reported the narrowing of the isthmus to be a recurrent anomaly in FAS, using objective manual measurements of callosal thickness and normative scaling analysis (Fraize et al., 2023c). We proposed to integrate it into an explicit compound neuroanatomical marker in the diagnostic decision tree. The ultimate goal is for neuroanatomy to contribute to clinical assessment, especially for subjects with non-syndromic forms.

To our knowledge, there is no fully automated reference tool for the measurement of the corpus callosum thickness, let alone automated analysis methods to tag and obtain individual measurements for singular points. Semi-automated measurement tools currently exist. For instance, Luders et al. proposed a technique beginning with manually delineating the corpus callosum on the sagittal midsection manually (Luders et al., 2006a). The upper and lower contours are then differentiated to compute the mean distance and generate the median line. The thickness of the corpus callosum is subsequently derived from this median line and compared at each interval. Following this pioneering study, the approach was implemented in the analysis of correlation with gender, IQ, and attention (Luders et al., 2006b, 2009, 2014; Westerhausen et al., 2011). Adamson et al. proposed another more automated method, albeit still involving human intervention (Adamson et al., 2011). Firstly, the two extremities are manually placed on the contour of the corpus callosum which is automatically generated. These two points are then adjusted to maximize the length of the center line. Subsequently, the center line is divided into 40 points. Finally, the thickness of the corpus callosum is determined by using either the orthogonality to the center line or by applying the Laplace equation. A fully automated method was proposed by Herron et al. based on a series of radial lines emanating from a centroid and intersecting the corpus callosum that are verticalized to unwrap the structure, define a median line and measure thickness (Herron et al., 2012; Li et al., 2017). The process relies on spatial normalization, the measurements being obtained in the *Montreal Neurological Institute* (MNI) space and transformed back to native anatomical space by inverting the affine spatial normalization transformation.

Regardless of how the thickness profile was obtained in these different studies (Sowell et al., 2001; Luders et al., 2009; Yang et al., 2012b; Danielsen et al., 2020), the subsequent analyses and comparisons required normalization into a common space. This normalization step is supported by a hypothesis, not always explicit, about how the callosal thickness varies as a function of the reference, either the full brain or the corpus callosum itself. It is also difficult to anticipate how these post-normalization analyses would accommodate incompleteness of the structure, for instance posterior agenesis for the corpus callosum, in terms of distortions or loss of signal, depending on the choice of normalization or template (Mangin et al., 2016). Normalization-based procedures and the underlying hypotheses are legitimate, but questionable when studying pathological populations with abnormally small brain or high risk of dysgenesis of the studied structure, which is the case with FASD and corpus callosum. At the very least, this encourages strategies of generation and analysis of the corpus callosum thickness profile that minimize the need for spatial normalization, above all spatial averaging, and enable explicit size effect models to be implemented. Indeed, the local size deficits must be interpreted by taking global brain size into account (scaling analysis) with a model that allows proportions to vary with brain size (allometric scaling). The relevance of the power law to describe the phenomena associated with brain size variations is firmly established (Toro et al., 2009; Germanaud et al., 2012; Liu et al., 2014; de Jong et al., 2017; Warling et al., 2021; Fraize et al., 2023b).

In this study, we first introduce a new method that is fast and fully automated, based on a first step of spectral analysis of the shape of the corpus callosum, which makes it possible to define and measure a continuous thickness profile at the individual level. We then propose a normalization-free strategy of analysis of this profile based on automatically defined local singular extrema. Lastly, we apply this spectral-based thickness profiling to a series of subjects with FASD and controls. We characterize brain size independent callosal anomalies in a fully automated manner, that were compared with the ones previously manually characterized in the same population (Fraize et al., 2023c).

## 2. Materials and methods

### 2.1. Participants

Eighty-nine consecutive subjects with FASD, aged 6 to 20 years, were retrospectively included from a clinical series of patients attending the dedicated child neurology consultation for neurodevelopmental disorders at Robert-Debré University Hospital (RD) between 2014 and 2020. FASD diagnosis was established on the basis of the guidelines of Astley (Astley, 2004) and a full differential diagnosis work-up was completed, including a systematic brain MRI. Individuals prenatally exposed to another embryo-fetotoxic agent were not included. FASD subjects were split into two groups: the syndromic FAS (including partial FAS) and the non-syndromic ones (NS-FASD). This series and the precise diagnostic procedure have already been described in previous studies (Fraize et al., 2023b,c). Clinical and radiological characteristics of the 52 FAS subjects (58.4%) and the 37 NS-FASD subjects (41.6%) are detailed in Table 1.

**Table 1:** Demographic, clinical, radiological data of FASD subjects.

	FAS *n* = 52	NS-FASD *n* = 37	FASD groups comparison *p*-value
*Sociodemographic assessment*			
Sex: male n (%)	27 (51.9)	25 (67.6)	0.209
Age at MRI, mean in years (SD)	10.93 (3.57)	11.88 (3.55)	0.219
*Clinical assessment, n (%)*			
*(1) Prenatal alcohol exposure*			
4.Confirmed, severe	21 (40.4)	16 (43.2)	0.959
3.Confirmed, moderate or unquantified	26 (50.0)	19 (51.1)	1.000
2.Not documented	5 (9.6)	2 (5.4)	0.748
1.No exposure	0 (0.0)	0 (0.0)	-
*(2) FAS facial features*			
4.Severe	31 (59.6)	2 (5.4)	**<0.001**
3.Moderate	21 (40.3)	1 (2.7)	**<0.001**
2.Mild	0 (0.0)	30 (81.1)	**<0.001**
1.None	0 (0.0)	4 (10.8)	0.057
*(3) Growth deficiency*			
4.Significant	19 (36.5)	3 (8.1)	**0.005**
3.Moderate	11 (21.2)	2 (5.4)	0.077
2.Mild	9 (17.3)	9 (24.3)	0.586
1.None	13 (25.0)	23 (62.2)	**0.001**
*Brain anatomy*			
(4) Structural central nervous system damage	40 (76.9)	19 (51.4)	**0.022**
Head circumference (smallest known)			
(4) ≤ − 2 SD: microcephaly	34 (65.4)	13 (35.1)	**0.009**

FAS, fetal alcohol syndrome; FASD, fetal alcohol spectrum disorder; NS-FASD, non-syndromic fetal alcohol spectrum disorder; SD, standard deviation. In bold, *p*-values < 0.05.

One hundred and twenty-six typically developing subjects, aged 6 to 20 years, with no report of PAE, developmental delay or family history of neurological or psychiatric condition (1^st^ degree) were included for comparison. A subgroup of 40 subjects was matched with the FASD group for the acquisition site (MRI scanner and sequence) as part of a research program on autism in the RD Psychiatry Department. Other typically developing subjects were part of previously published studies (Germanaud et al., 2014; Bouyeure et al., 2018).

There were no significant differences in the control group compared to the FASD group for sex (50.8% vs. 58.4% of males respectively, *p* = 0.334) and age at MRI (12.08 vs.11.32 years of age respectively, *p* = 0.116).

This study was conducted in accordance with the principles of the Declaration of Helsinki. Subjects’ data were studied in accordance with French regulation (MR-004, declaration of conformity n°2059980v0), following approval by the Paris-Saclay research ethics committee (CER-Paris-Saclay-2020-094). Controls’ data were used within the framework of the ethical authorizations of the primary studies (Gene and autism, Inserm C07-33, 08–029 and 11–008).

### 2.2. MRI data

For both FASD subjects and 40 site-matched controls, MRI acquisitions were performed in the Department of Pediatric Radiology of RD Hospital at 1.5 T (Ingenia, Philips Healthcare, Amsterdam, the Netherlands) with a 3DT1 FFE-TFE sequence (1 mm isotropic; TR = 8.2 ms; TE = 3.8 ms; TI = 0.8 s; Flip = 8°; SENSE = 2). Another group of 31 controls was acquired at the Frédéric Joliot Hospital (SHFJ, CEA-Saclay) at 1.5 T (Signa, GE Healthcare, Milwaukee, US) with a 3DT1 GE FSPGR sequence (1x1x1.2 mm; TR = 9.9 ms; TE = 2 ms; TI = 0.6 s; Flip = 10°) and a third one of 55 controls subjects on a 3 T (Siemens Trio, Siemens Healthineers, Oxford, UK) at NeuroSpin (NS, CEA-Saclay) with a 3DT1 Siemens MPRAGE sequence (1 mm isotropic; TR = 2.3 s; TE = 3 ms; TI = 0.9 s; Flip = 9°; GRAPPA 2). A visual quality check was systematically performed (JF, DG) to exclude images of insufficient quality.

### 2.3. MRI processing

#### 2.3.1. Total brain volume

The total brain volume was obtained by *volBrain* (Manjón and Coupé, 2016), and was highly correlated (*R*^2^ = 75%) with the previously used proxy of brain size, that is, the axial reference brain surface (Supplementary Figure 1; Fraize et al., 2023c).

#### 2.3.2. Midsagittal section of the corpus callosum

The mask of the midsagittal section of the corpus callosum was obtained within the *Morphologist2015* framework of *BrainVisa*^1^ after oversampling of the T1-weighted images at 0.5 mm isotropic resolution. This section was localized in the interhemispheric plane by intersecting the white matter mask with the midsagittal plane in the Talairach space. The mask was checked systematically for any obvious segmentation error that could be manually corrected (Supplementary Table 1).

#### 2.3.3. Spectral analysis of the shape using the Fiedler vector

**Figure 1:**
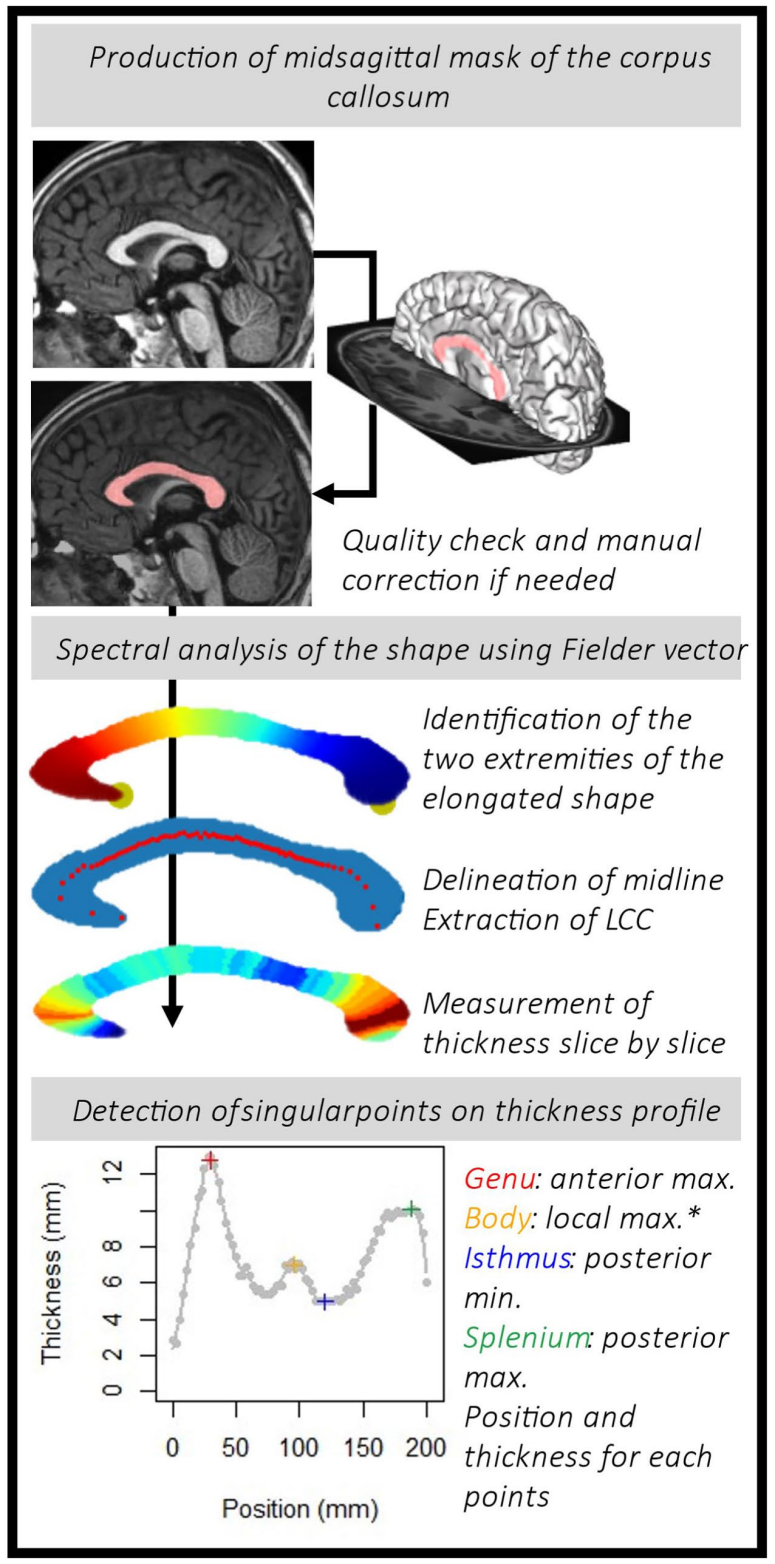
Pipeline to obtain the thickness profile for each subject and identify the points of interest: the genu, the body hump (*see details in the text and in Figure 3), the isthmus and the splenium.

From the mask of the midsagittal section of the corpus callosum, we built a 26-connectivity graph. To do so, we considered the 3D points from the one row of voxel of the mask and added edges among the 26 possible neighbors if belongs to mask. We used the Fiedler vector of this graph, the first non-trivial eigenvector of the Laplacian matrix, to compute a quasi-isometric parameterization (Lefevre et al., 2012; Coulon et al., 2015). This mathematical tool tracked the main elongation vector and identified extremal points. We added a simple algorithm to reposition the rostrum so that its position is correct (see details in Supplementary Figure 2). The Laplacian graphs were perturbed to create a ‘new’ Fiedler vector that followed the correct elongation (Lefèvre et al., 2023). From this ‘new’ Fiedler vector, we calculated isolines (equivalent value). Then, regular bins were defined between the min and max values of the Fiedler vector. It provided slices of the shape, of irregular width. The barycenter of each slice was computed to form the midline (see the irregularly spaced red dots on the Figure 1). From this midline and reparametrized vector, we obtained new isolines and slices of constant and regular thickness. Using PCA within each slice, the main direction, roughly orthogonal to the median line, was used to extract regular thicknesses. We finally obtained a map of the corpus callosum thickness on regularly and optimally spaced slices (between 50 and 80) following the longitudinal orientation of the shape. The code used to perform spectral analysis of the corpus callosum midsagittal section is released on https://github.com/JulienLefevreMars/CorpusCallosumParameterization.

#### 2.3.4. Thickness profile and singular points

The first parameter of interest extracted was the length of the corpus callosum (LCC) obtained from the length of the curvilinear abscissa between the two extreme points. Then, we targeted the local singular extrema on the continuous thickness profile curve fitted with cubic smoothing spline (smooth.spline() R function). The smoothing parameters were set at the same level for each subject. *Spar* is the smoothing parameter controlling the trade-off between fidelity to the data and roughness of the function estimate. It is derived from the lambda parameter, scaled between 0 and 1. Here, we fixed the *spar* parameter at 0.4. To characterize the morphology of the corpus callosum, radiologists and clinicians usually measure the thickness of the genu at the thickest point anteriorly, the body (local thickness), the isthmus at the thinnest point posteriorly and the splenium at the thickest point posteriorly (Garel et al., 2011; Fraize et al., 2023c). From the thickness profiles, we extracted the positions and the thicknesses of the genu (GT) and the splenium (ST), the local maximums of the two extremities, and of the isthmus (IT), the local minimum between these two points. In addition, we looked for localized thickening, a hump, within the body (Figure 1). As the amplitude of this thickening could potentially be small and to make sure that it was independent of curve smoothing variations, we sought it on the thickness profile curve between the genu and the isthmus, by varying the level of smoothing (10 levels in addition to the one initially chosen, from 0.2 to 0.7). To locate the body hump, we identified the local maximum(s) whose position was consensual on the curvilinear abscissa on more than 8 of the smoothing levels. We extracted the body hump thickness (BHT) or the mean thickness if there were several humps (no more than two). We also extracted the median of the thicknesses between the genu and the isthmus, as the median body thickness (MBT). All these processing steps are summed up in Figure 1.

These length and thickness parameters were correlated with available gold standard manual measures using the PACS measurement tools (Carestream, New York, NY, USA) (Fraize et al., 2023c) by establishing the coefficient of determination R^2^ and the mean percentage error (MPE = mean of the difference between manual and automated measurements divided by manual measurement).

### 2.4. Modeling and statistical analysis

Statistics were performed using R Project for Statistical Computing (RRID: SCR_001905), with a 5% alpha risk. A False Discovery Rate (FDR) method was systematically applied to correct for multiple comparisons (Benjamini and Hochberg, 1995) at each analysis step (all parameters together). Group differences in clinical characteristics and parameters were evaluated using two-sample *t*-tests for continuous variables, and a Chi-square test for categorical variables, ANOVA for group effect. To assess the normality assumption of the t-tests and the ANOVA, we first performed the Kolmogorov–Smirnov test for each of the 3 groups, and the value of *p*s were not significant for any of the groups. A value of *p* is significant when the data do not appear to be normally distributed.

Since the data were acquired on 3 sites, we adjusted for batch effect prior to analyses using ComBat, a method based on an empirical Bayes framework (Johnson et al., 2007; Fortin et al., 2018), integrating into the model sex, age, and diagnosis as covariates to be spared, considering their possible interaction (Supplementary Table 3). We then performed unifactorial group comparisons between FASD and controls knowing that these groups were matched for age and sex. First we compared the whole thickness profile after rigid length normalization to provide a continuous quantitative comparison of the radiological aspect of the corpus callosum. Second, we compared the five parameters previously defined (callosal length and the thickness of the 4 singular points). Lastly, we implemented clinically inspired normative analysis to provide an individual-based comparison of these five parameters, accounting for two major covariates, i.e., age and brain size: first a normative growth analysis (age) then a normative scaling analysis (brain size).

#### 2.4.1. Unifactorial group comparison

We compared the thickness profiles of the controls with those of the two FASD groups. After rigid spatial normalization in the curvilinear abscissa axis, we oversampled thicknesses along this normed axis (*n* = 100) then adjusted for site effect. We studied the effect of FASD, using a linear mixed model considering diagnosis (controls vs. FAS or NS-FASD) as a fixed effect and random effect for each subject to account for subject-specific variability. Nonparametric cluster-based permutation analyses were carried out using a method inspired by EEG-MEG analysis to locate and group zones of significantly different thicknesses for spatial consistency (originally, time-points with temporal consistency in EEG signals), considering corrections for multiple comparisons (Maris and Oostenveld, 2007). Significance probability was calculated using the cluster-mass statistic (cluster-forming alpha = 0.05, randomizations = 1,000).

#### 2.4.2. Normative analyses

To define the growth curves, the relationships between measured parameters (LCC and thicknesses at singular points adjusted for site effect) and age were modeled in the control group. We applied a general additive model considering homoscedasticity over the age range to fit the model and obtain percentiles of the distribution (Wood and Fasiolo, 2017; Dinga et al., 2021).

To define the scaling curves, the relationships between measured parameters (P) and brain size (total brain volume, TBV) were modeled in the control group, by a power law with scaling coefficient a and constant b (P = b × TBV^a^) to take into account expected allometric effects (changes in proportions with size) (Liu et al., 2014; de Jong et al., 2017; Warling et al., 2021). Using bootstrap resampling and empirical quantiles, we obtained the unbiased prediction intervals (90th and 10th percentiles) (Guenther, 1971; Lee and Scholtes, 2014).

For each parameter, FASD subjects with measurements below the 10^th^ percentile curve were counted as normatively too small for age or for brain size. An abnormality was considered recurrent when the number of too-small measurements in the FAS or NS-FASD group exceeded the theoretically expected 10% of the individuals (binomial test).

## 3. Results

### 3.1. Identification of singular points

**Figure 2:**
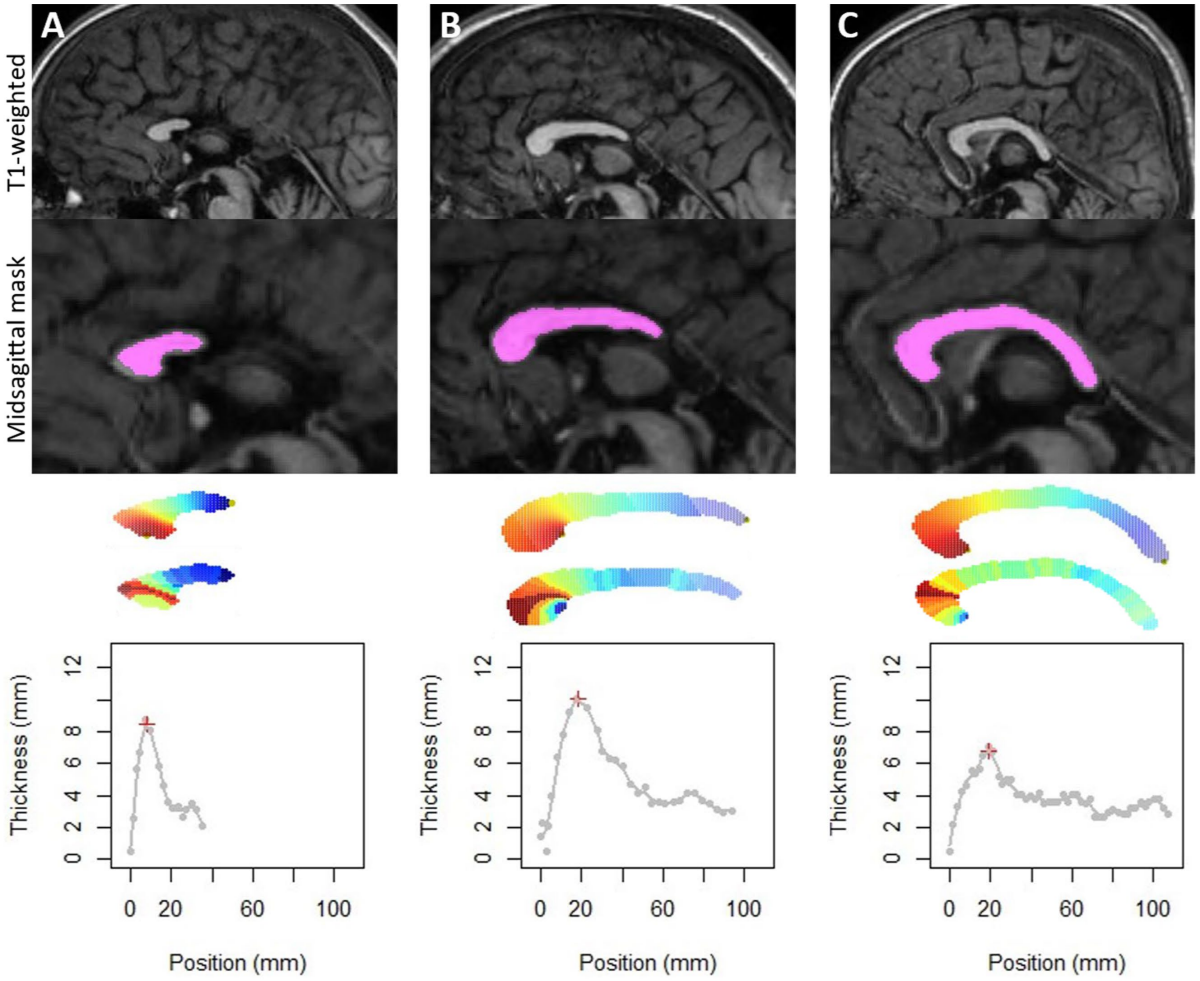
Application of the method on the three FAS subjects with partial agenesis **(A,B)** or with too thin corpus callosum **(C)**, which could also be considered as a very posterior agenesis of the splenium. Note that our method detects the longest elongation and allows us to obtain thicknesses by slice. On the other hand, detection of singular points was not possible.

The method was successfully applied to all subjects. For the two FAS subjects with posterior agenesis and the one with an obvious posterior thinning, the thickness profile was also extracted and analyzed (Figure 2). They were not included for further analysis as they were obvious outliers with missing singular points.

From the thickness profiles, we were able to identify for each subject the position and thickness of the genu, isthmus and splenium automatically.

**Figure 3:**
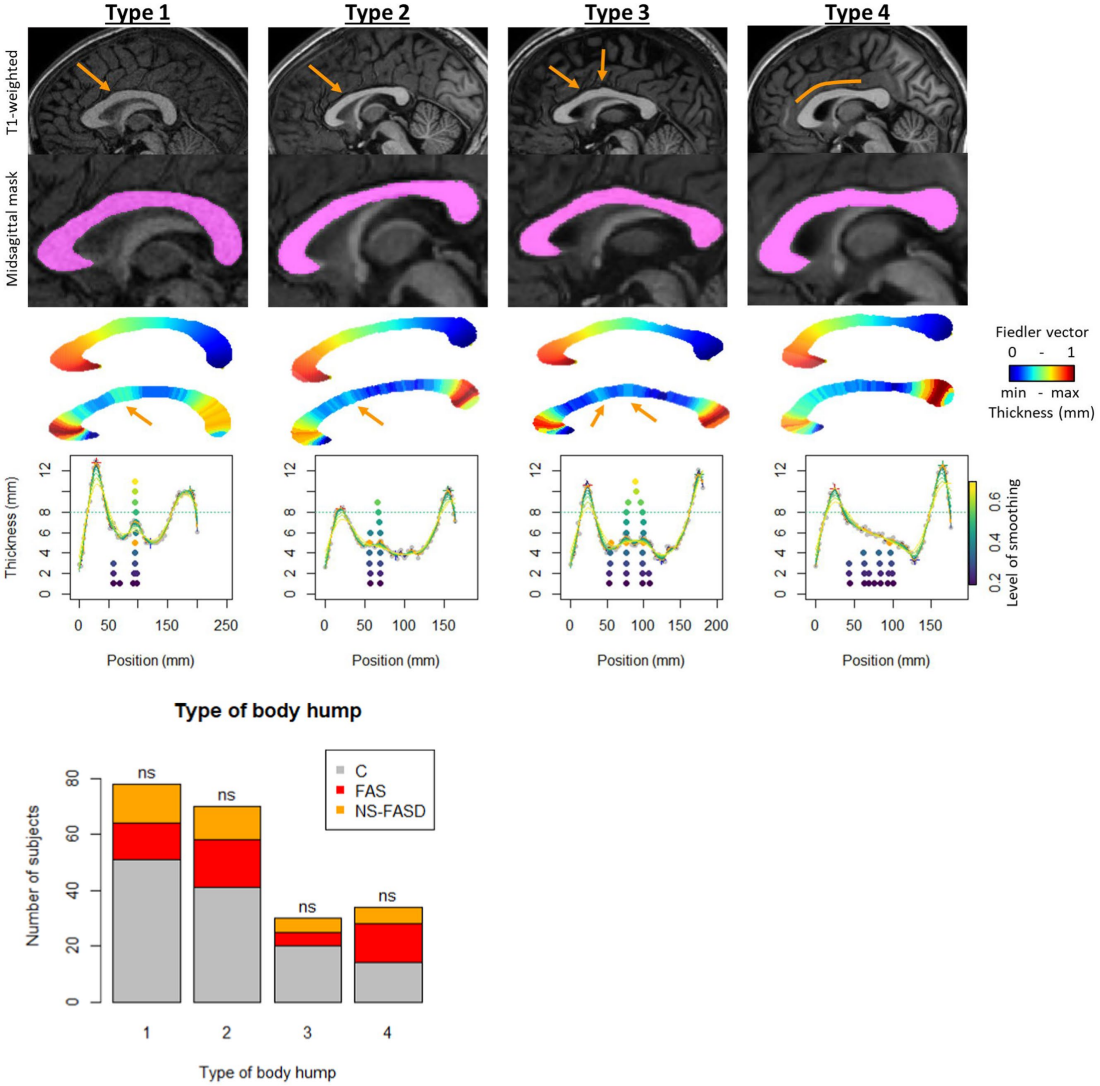
Body hump identification. Example of subject by group, variation of smoothing level to identify local maximums. First row: T1 anatomical image with location of the hump (orange arrow). Second row: the corpus callosum mask on sagittal midsection. Third row: Fiedler vector value (posterior–anterior gradient from 0 to 1). Fourth row: slice thickness (scaled gradient from red greatest to smallest blue thickness). Fifth row: thickness profile as a function of position along the curvilinear abscissa. Variation in smoothing level (colored curves) and position of local maximums between genu and isthmus, from lowest smoothing level (bottom dark purple point) to highest (top light-yellow point). Type I: position of the body hump obvious and consensual for all smoothing levels. Type 2: position of the body hump obvious and consensual for more than 8 smoothing levels. Type 3: two body humps obvious and consensual for more than 8 smoothing levels. Type 4: no body hump. Distribution of types by group: control (C) and FASD (FAS and NS-FASD). Comparison of proportion of type between groups. ns: f not significant, value of *p* < 0.05; value of *p*s were adjusted for multiple comparisons using the FDR method.

We divided the subjects into 4 types to define the presence or not of the body hump. In type 1, the position of the body hump was obvious and consensual for all smoothing levels, accounting for 78 subjects (36.8%). In type 2, the position of the body hump was obvious and consensual for more than 8 smoothing levels, accounting for 70 subjects (33.0%). In type 3, two body humps were obvious and consensual for more than 8 smoothing levels, accounting for 30 subjects (14.2%). In type 4, no body hump was identified, accounting for 34 subjects (16.0%) (Figure 3).

The BHT was thus only considered for type 1, 2 and 3 subjects (*n* = 178).

**Figure 4:**
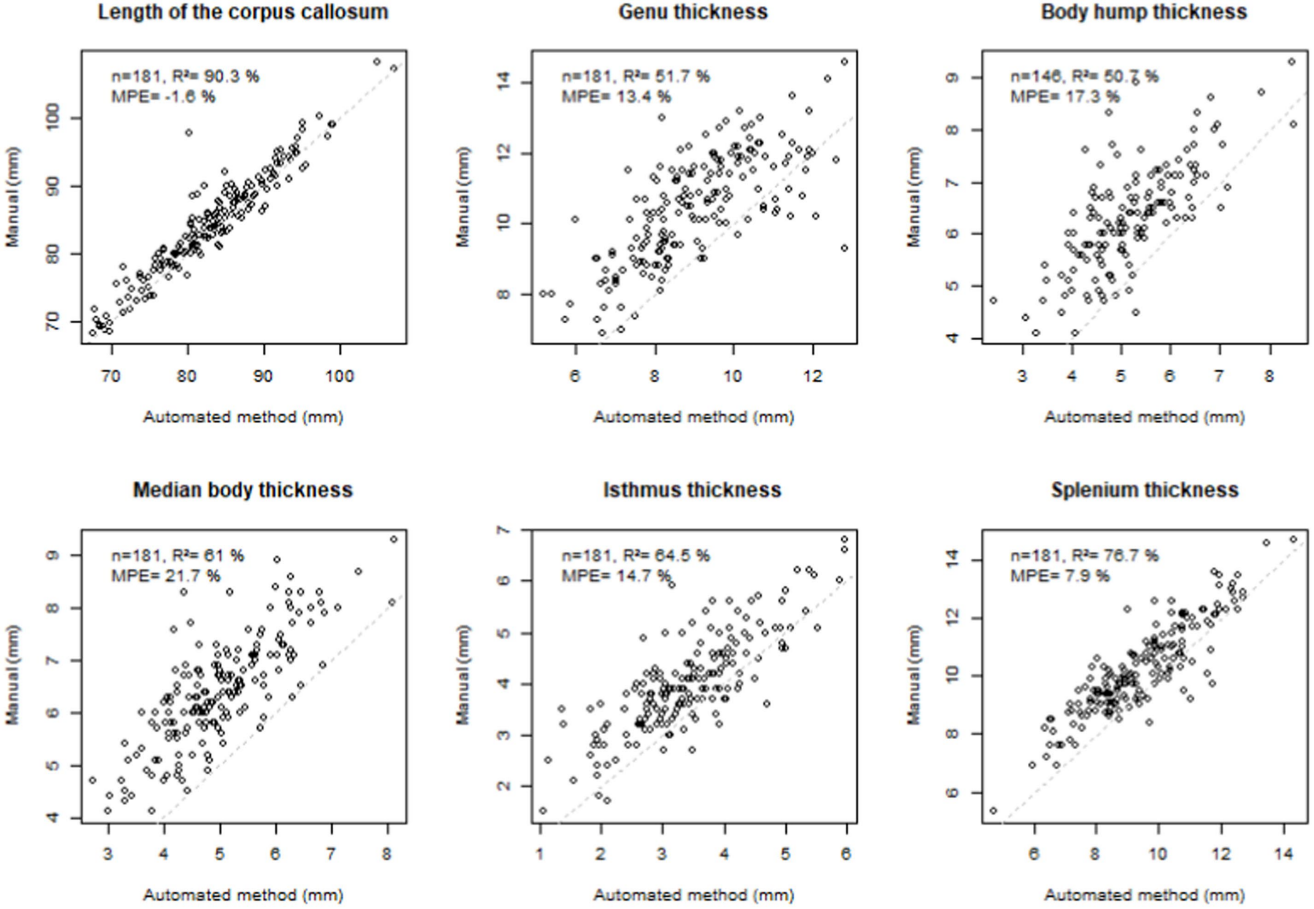
Comparison with manual measurements. Top left: number of subjects (*n*), coefficient of determination (*R*^2^), mean percentage error (MPE = mean of the difference between manual and automated measurements divided by manual measurement), correlation line 1 to 1 in grey dotted line. Note that, *n* = 181 corresponds to the total number of subjects who had manual measurements in the previous study (Fraize et al., 2023c, excluding the subgroup of 3T controls), *n* = 146 corresponds to the total number of subjects who had manual measurements in the previous study and had hump with type 1–3. Agenetic subjects were excluded.

The measurements obtained by our method were strongly correlated (*R*^2^ > 50%), with the gold standard of manual measurements, the most highly correlated being that of the LCC (*R*^2^ = 90.3%) (Figure 4). For this measurement, the error was minimal (mean percentage error MPE = 1.6%). This error was moderate for the isthmus, genu and splenium but systematically underestimated (positive MPE < 15%). The manual location for the body thickness measurement (at the central point) did not directly correspond to the measurements collected in the current study, resulting in larger MPE (~ 20%).

### 3.2. Control and FASD comparison

#### 3.2.1. Unifactorial comparison of thickness profiles

**Figure 5:**
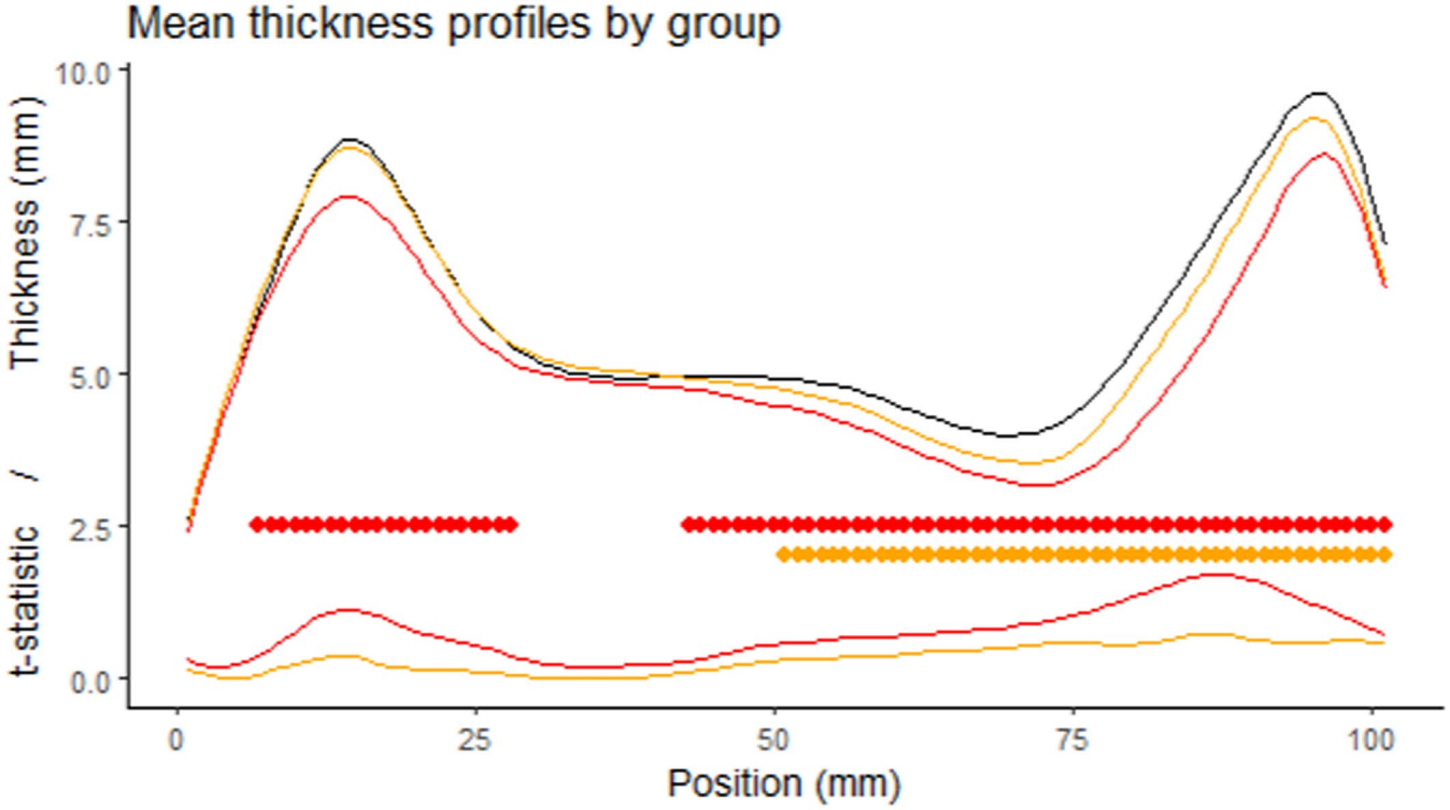
Comparison of the thickness profiles. First row: mean profile of the control group in black (*n* = 126), the FAS group in red (*n* = 52), the NS-FASD group in orange (n = 37), after rigid length normalization. Second row: cluster of significantly different thicknesses (controls vs. FAS in red or NS-FASD in orange, value of *p* of cluster mass statistic <0.05, including adjustment for multiple comparisons). Third row: effect size (*t*-statistic of the linear mixed model).

Comparing the thickness profiles of control and FAS subjects after rigid length normalization, we identified two significantly different sections, one around the genu and one consisting of the whole posterior half but with a peak in effect size in the anterior part of the splenium. For NS-FASD, the zone of significance was less extended, and limited to the posterior half (Figure 5).

#### 3.2.2. Unifactorial comparison of length and thickness of singular points

There was no significantly different proportion of body hump type between the FASD group and the control group. The distribution of types by group is detailed in Figure 3.

**Figure 6:**
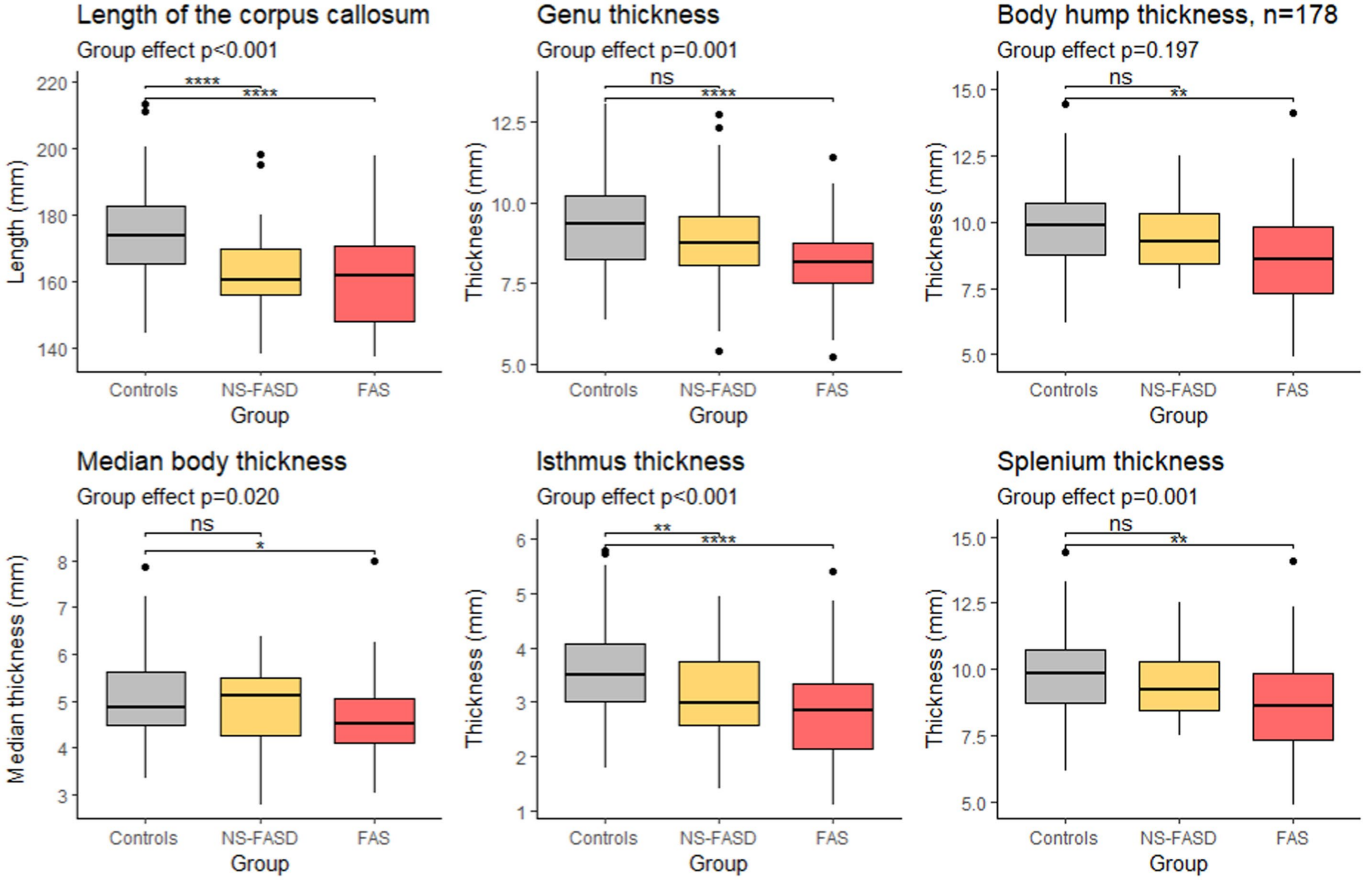
Comparison of length and thicknesses of singular points. Group effect (control, FAS and NS-FASD) on ANOVA. Top bracket, value of *p* of *t*-test of controls versus FAS or NS-FASD. ns: *p* > 0.05, *p ≤ 0.05, **p ≤ 0.01, ***p ≤ 0.001, ****p ≤ 0.0001. Value of *p*s were adjusted for multiple comparisons using the FDR method.

A significant group effect was found for the length of the corpus callosum, the genu, the median body, the isthmus, and the splenium thicknesses. The difference between the control group and the FAS group was significant for all parameters. The difference between the control group and the NS-FASD group was significant only for the length of the corpus callosum and the isthmus thickness (*p* < 0.05) (Figure 6).

### 3.3. Normative analysis

#### 3.3.1. Growth charts

**Figure 7:**
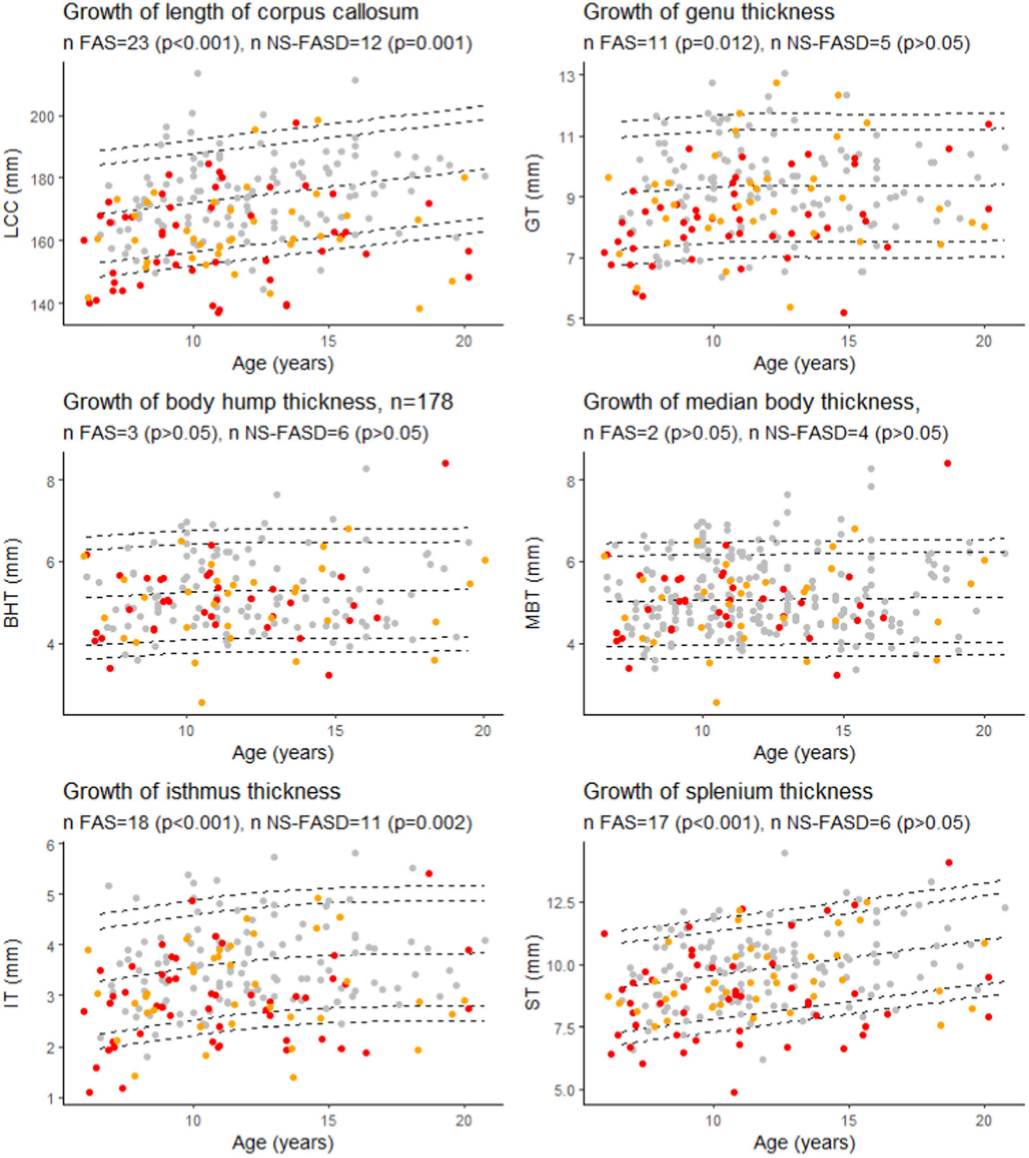
Growth charts for length of the corpus callosum (LCC), the genu (GT), the isthmus (IT), the body hump (BHT), the median body (MBT), and the splenium (ST) thicknesses. Number of fetal alcohol spectrum disorder subjects under the 10th percentile (value of *p* of binomial test, value of *p*s were adjusted for multiple comparisons using the FDR method). Controls in grey, FAS in red, NS-FASD in orange. The 95, 90, 10 and 5th percentiles are represented in dotted lines.

There was an excess of FASD subjects below the 10th percentile for age for the length of the corpus callosum, the isthmus, and only for FAS subjects for the genu and the splenium (*p* < 0.05) (Figure 7).

#### 3.3.2. Scaling charts

**Figure 8:**
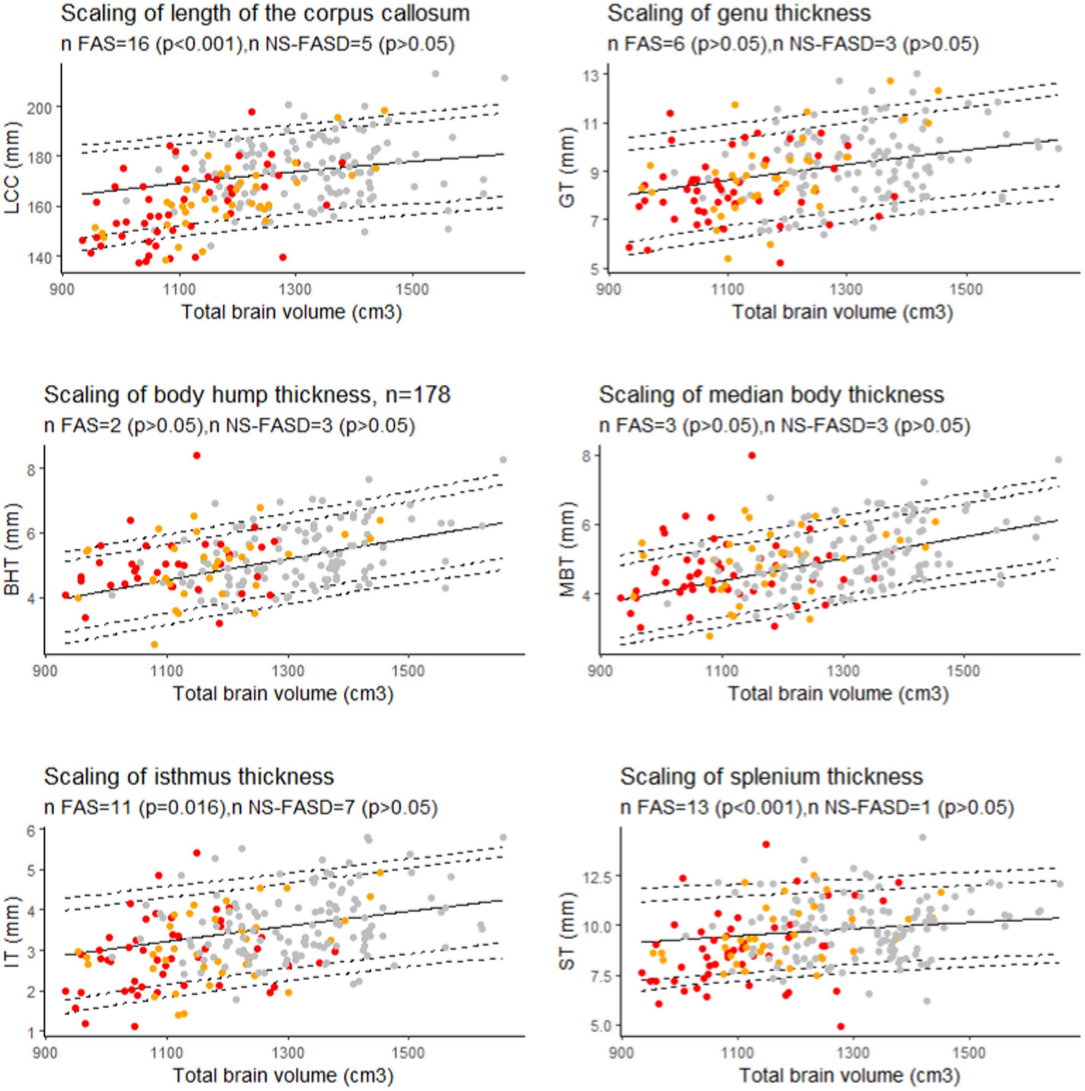
Scaling charts for length of the corpus callosum (LCC), the genu (GT), the isthmus (IT), the body hump (BHT), the median body (MBT), and the splenium (ST) thicknesses. Number of fetal alcohol spectrum disorder subjects under the 10th percentile (value of *p* of binomial test, *p*-values were adjusted for multiple comparisons using the FDR method.). Controls in grey, FAS in red, NS-FASD in orange. The 95, 90, 10 and 5th percentiles are represented in dotted lines.

There was an excess of FAS subjects below the 10th percentile for brain size for the length of the corpus callosum, the isthmus, and the splenium (p < 0.05). NS-FASD subjects were not over-represented below the 10th percentile for brain size (*p* > 0.05) (Figure 8).

## 4. Discussion

In this study, we introduce the first fully automated, normalization-free, fast method to produce and analyze the thickness profile of the corpus callosum. We applied it to the morphometric comparison of 89 subjects with FASD to 126 typically developing controls, aged between 6 and 20 years old. We were able to show an apparent downsizing of the corpus callosum both in length and thickness in FAS, predominating in the posterior half and less pronounced in NS-FASD. We established that a recurring abnormal undersizing was observed in FAS only for the length and the isthmic and splenial thicknesses when controlling for brain size. Thus, these results with a new and convenient automated method not only replicate, but also extend our previous results with manual measurements including the same dataset, that were only able to show recurrent isthmic anomaly (Fraize et al., 2023c).

### 4.1. Confirmed and newly revealed differences between FASD and controls

#### 4.1.1. What can be observed on native measurements?

After rigid linear normalization and adjustment for site, native thickness profiles showed extensive but not homogeneous thinning of the corpus callosum in FASD. It was reduced in the posterior half section in both FAS and non-syndromic FASD subjects and reduced in the genu section only in FAS subjects (Figure 5). The rigid anteroposterior deformation coarsely aligns the thickness profiles of each subject, enabling an initial average assessment of what is visible to the radiologist in this population. It points to the posterior zone with a possible additional damage of the genu in FAS and lower severity in NS-FASD.

Considering the native thickness of the four singular local extrema (genu, body hump, isthmus, and splenium), we observed significant differences between the FAS and the control groups for all these extrema and between the NS-FASD and the control groups for the isthmus only (Figure 6). However, the effect on body thickness appeared much smaller, and was not significant when the 3 groups were compared together (ANOVA). In addition, we showed no impact of FASD on body hump morphology, since there was no significant difference in the proportion of body hump type between groups (*p* < 0.05) (Figure 3). A recent study has evoked and dismissed the over-representation of “notching” among subjects with FASD (Schneble et al., 2020), in the medico-judicial context of FASD recognition, but using a very qualitative assessment. Our results are consistent with the fact that the body and its possible hump(s) are spared from callosal thinning in FASD, which may be seen as clinically relevant *per se*.

We also showed that the overall length of the corpus callosum was reduced (*p* < 0.001) on average, with no other adjustment than site effect, in both NS-FASD and FAS (Figure 6). All these results are consistent with previous studies describing a smaller corpus callosum (Riley et al., 1995; Sowell et al., 2001; Astley et al., 2009; Biffen et al., 2017; Jacobson et al., 2017; Fraize et al., 2023c) and pointing out the posterior part as the most affected one (Sowell et al., 2001; Yang et al., 2012a; Fraize et al., 2023a). Yet, even if our FASD and control groups were matched for age and sex, there were inherent differences in brain size, both age and size having potentially nonlinear effects to be accounted for in further analyses.

#### 4.1.2. What remains at the individual level after independent control of major covariates?

Since birth, the shape of the corpus callosum changes as revealed by morphometric parameters (Rajapakse et al., 1996; Westerhausen et al., 2011; Herron et al., 2012). Within the increasingly promoted and clinically relevant framework of normative analyses, we proposed to take age into account on the basis of developmental growth charts derived from our typically developing control sample. We found FAS-recurrent anomalies (excess of FAS subjects below the 10th percentile) for the LCC, the genu, the isthmus, and the splenium thicknesses (Figure 7). This means that considering the age effect did not modify our previous description of the large downsizing of the corpus callosum both in length and thickness in FAS, predominating in the posterior half and in the genu. This minor effect of age on the measurements is consistent with asymptotic linear low (length, splenium) or null (other thicknesses) residual growth of the corpus callosum after 6 years already described in the typically developing pediatric population (Garel et al., 2011; Fraize et al., 2023c).

In the FASD population with brain growth deficiency (Archibald et al., 2001; Astley et al., 2009; Rajaprakash et al., 2014; Treit et al., 2016; Boronat et al., 2017), brain size must be properly considered when interpreting other neuroanatomical measurements. Thus, to describe the reduction of the corpus callosum measurements observed in FASD at the individual level, we added to the classical normative analysis on growth charts (effect of age) the analysis on scaling charts (effect of size). We ensured that the scaling model fitted in the control group correctly projected to the smaller FASD range of brain sizes by using a biomathematical model that captures the gradual change in proportions along size range. This allometric scaling was modeled by a power law whose relevance and interest have been widely argued and documented (Germanaud et al., 2014; Liu et al., 2014; de Jong et al., 2017; Warling et al., 2021). Itis already used in anatomical descriptions of the corpus callosum in newborns (Lewis et al., 2022). Hence, using allometry-sensitive scaling charts to account for brain size effect, we showed that thinning (below the 10th percentile) remained recurrently abnormal in FAS only for the isthmus and the splenium thickness (Figure 8). We additionally showed a significant excess of FAS subjects with undersized total LCC for their brain size (Figure 8). So, we confirmed and extended the results of our previous study (Fraize et al., 2023c), by adding both brain size independent anterio-posterior length and splenium thickness impairments to that of the isthmus. This involvement of the splenium is also consistent with our previous findings showing a reduction of its posterior half surface independent from hemispheric size (cortical surface) in a subsample of subjects (Fraize et al., 2023a).

These new findings may be explained by the greater number of controls (*n* = 126 vs. 94), the more reliable estimate of brain size (total brain volume vs. surface of a reference brain area), the more elaborate model of regression and the more robust assessment of percentiles (Fraize et al., 2023c). The addition of the ComBat batch effect adjustment could also have improved sensitivity (see Supplementary Table 2). Previously, there was no significant site effect to be corrected in the manual measures (Fraize et al., 2023c). In this case, *post hoc* analyses without adjustment for batch effect showed no difference in the present results (data not shown). It is also possible and a reasonable assumption that automated measurement of thickness of the splenium is more accurate and less noisy, enabling more discriminating statistical comparison.

#### 4.1.3. Potential neuroanatomical markers of FASD in corpus callosum

We did not find any size-independent recurrent anomalies taken one by one in our small NS-FASD subgroup. But for these subjects we have already proposed a perspective of cumulative diagnostic probability for diagnostic improvement (Fraize et al., 2023c). Arguably, the likelihood of the causal link with alcohol exposure increases for NS-FASD subjects with one or more anomalies found to be recurrent in FAS in the final scaling analysis (LCC, IT, ST, in Figure 8). In this case, these new results in FAS could provide additional diagnostic arguments and ease the way to more confident diagnoses in certain subjects presenting FASD without FAS.

Yet, the posterior damage of the corpus callosum with preservation of the body is probably not specific to FASD, as callosal anomalies are not rare in the general (Glass et al., 2008) nor in the neurodevelopmentally disabled population (Jeret et al., 1985). But we have already argued that the entire corpus callosum morphology could constitute a radiological signature likely to increase diagnostic certainty, particularly in NS-FASD. Moreover, other focal neuroanatomical anomalies such as cerebellar damage (O’Hare et al., 2005; Cardenas et al., 2014; Sullivan et al., 2020; Fraize et al., 2023b) or deep grey nuclei volume reduction (Nardelli et al., 2011; Roussotte et al., 2012; Nakhid et al., 2022), should also be considered in a composite, multidimensional neuroanatomical score reflecting fetal alcohol-induced brain dysmorphia.

### 4.2. New method for analyzing the shape of the corpus callosum

Our new method for corpus callosum shape analysis was developed to automatically detect the anterior point of the rostrum and decide on a posterior point of the splenium. It is advantageous over the existing ones because it does not require human intervention (Luders et al., 2006b; Adamson et al., 2011) and does not involve spatial normalization (Herron et al., 2012). In its current state, only the existence of a rostrum is considered. Our method is based on the maximum elongation of this two-dimensional shape, so it can accommodate any posterior shape of the corpus callosum. The three cases of agenesis or incomplete corpus callosum prove that this method is robust to variation of corpus callosum shape, even pathological (Figure 2), at least posterior ones. In these agenetic cases, we did not integrate the detection of singular points, which are not consensual and would require human supervision. For all subjects, this method produced continuous thicknesses along the entire length of the corpus callosum, and unequivocally detected singular local extrema at singular points (the genu, the hump of the body, the isthmus, and the splenium). Extracted thickness measurements were highly correlated with the gold standard manual measurements (Figure 4), despite a small to moderate underestimation (MPE < +15%). The two methods deal with partial volume effects on the edges differently. The manual process relies on the visualization smoothing included in the PACS viewer that could potentially biased the eye of the expert. The automated one relies on the segmentation of the mask of the corpus callosum – based on the contrast between grey matter, white matter and CSF – providing a skinnier or even eroded profile, logically resulting in a higher MPE for small measures than for large ones (genu, body, isthmus). Bear in mind that noise could also be induced by inaccuracy of the thinnest measurements close to the resolution of the oversampled mask (0.5 × 0.5 mm). Finally, even if manual measurement is the gold standard, the automated method may be more objective, reproducible, and less sensitive to global brain shape variation.

The first step, i.e., producing the mask of the corpus callosum on the sagittal section, may seem trivial but is in fact a decisive one. In our pipeline, this step was based on robust intensity-based *Morphologist* segmentation bricks, but a quality check was systematically performed, and manual corrections were sometimes necessary. Our method could benefit from the use of other software able to provide the 2D voxel-based mask, for example *Freesurfer* (actually 3D) or any new and specific methods (Adamson et al., 2014; Herrera et al., 2022), without modifying the subsequent steps. Ultimately, although not all steps are encapsulated and seamlessly connected, our new tool offers the advantage of having extremely short and computationally inexpensive steps. As a result, the cumulative computation time per subject to generate numerical parameters is less than 5 min.

On a methodological aspect, the correction of the Fiedler vector to reposition the rostrum is an example of partially-supervised information (*a priori*-based) in an otherwise unsupervised context (Lefèvre et al., 2023). It may be possible in the future to add other kinds of information, of various kinds (lines), to add constraints on the Fiedler vector or more generally on the Laplacian modes. More broadly, the developed methodological tool based on the Fiedler vector opens the way to other fields of characterization of the shape of the corpus callosum, such as the circularity, the surface per slice, or deformation per section, which could be the object of further study (Coulon et al., 2015; Walsh et al., 2019).

For now, as far as we know, this is the only fully automated method without any human intervention and normalization step for obtaining corpus callosum thicknesses. In fact, its use could go far beyond FASD and be applied in other pathologies where the morphology of the corpus callosum needs to be described.

### 4.3. Translating to daily clinical practice

In this study, we aimed to establish an analysis protocol that would ultimately be compatible with translation to clinical practice. The ultimate goal is to switch from manual measurements, which may be imprecise and noisy if non-expert, to secure computational automated and fast measurements, which could be facilitated by a computer interface. The proposed tool and analysis protocol should be acceptable in clinical practice because the produced measurements correspond to those already made in clinical settings (Garel et al., 2011; Ambrosino et al., 2022; Fraize et al., 2023c), and because their anatomical positioning is probably more objective. Acceptability should also be promoted by the fact that it leads to a normative analysis and therefore to categorial (normal, abnormal) information at the individual level.

Based on easy-to-use scaling charts and a proper biomathematical scaling model, we propose a method for detecting subject-level abnormalities, even with the partial overlap of brain size between patients and controls. However, the choice of reference population and adequacy of the control group raise questions. Our study included 126 typically developing individuals matched for age and sex, with no prenatal exposure. The automated segmentation, profiling, and singular local extrema detection enable the use of large imaging databases to establish more reliable charts, potentially stratified by age and sex. Despite the need to address site effects, our results show that a population of over a hundred controls currently allows for the establishment of informative normative charts.

### 4.4. Identification of a singular thickening of the body section in the general population

Our method leads to the description of a possible anatomical particularity of the corpus callosum, in FASD and more generally in healthy subjects. We identified a local thickening of the body section, either single (~70%) or double (16%) in most of the subjects (Figure 4). This local maximum of the thickness profile between the genu and the isthmus was automatically identified by using multi-scale-smoothed profiles inspired by scaled space implementation detecting robust maxima through a range of smoothing levels. This is almost equivalent to applying an amplitude limit without having to quantify the hump in mm in subjects with varying CC sizes. The literature on this morphological feature in the general population is sparse (Krause et al., 2019; Simpson et al., 2020). Krause et al., reviewed MR images of the corpus callosum of over 1,600 healthy subjects and denominated concavities in the dorsal surface “undulations” and “notching.” They found 50% of subjects with one notch and around 10% with two, and therefore possibly equivalent proportions of localized thickening.

We decided to add the median of the body thickness for all subjects, to compensate for the lack of consensus on the actual existence of this hump and the absence of an exact definition for the location of the body thickness measurement (Garel et al., 2011),. This corresponds to the median value of the thicknesses on the section between the genu and the isthmus. Although it was somewhat arbitrary, it correlated well with the manual measurement (*R*^2^ = 61%). In practice, and even if this is a finalist argument, the results were ultimately consistent between the two measures.

### 4.5. Other limitations and future directions

One limitation of the proposed modeling for analyses is the lack of consideration for the effect of sex (Ardekani et al., 2013; Lewis et al., 2022). We made this choice on the hypothesis that the downsizing of the control sample by a factor of two to establish sex specific charts would have lowered the statistical power of our study too much. We would have increased the risk of missing results, even with a reduced residual variance due to better adequacy of the modeling. It is a questionable choice that we also made and argued for our previous study, showing that results were almost unaffected (meaning that with this population size, the two choices may probably be equivalent). Yet as mentioned previously, with larger databases being available, it will be more efficient, even with clinical applications in mind, to establish differentiated female/male referential charts. It could also very well lead to the description of differential FAS-related abnormalities and markers between males and females (Chen et al., 2011; Zhou et al., 2018; Little and Beaulieu, 2020; Treit et al., 2020).

The limitations associated to our cohort of FASD subjects are multiple: retrospective data, absence of quantitative data on the level of exposure, over-representation of FAS subjects. But we would like to confirm, since this is not possible or recommended in all countries, that in our practice, brain imaging is systematic. There is no predictable bias in severity or clinical representation related to MRI availability.

## 5. Conclusion

Our study is the first to propose a fully automated tool to assess the corpus callosum thickness profile along its curvilinear abscissa and analyze it through the identification of 4 singular points. We used it to describe the callosal damage in a large cohort of FASD. We confirmed the excessive thinning of the isthmus for brain size. We revealed the global excessive shortening of the corpus callosum and thinning of the splenium for brain size to be recurrent anomalies in FAS subjects. The introduction of this tool in clinical practice is at hand and our study completes the knowledge on FASD callosal damage and reinforces the conviction that a neuroanatomical signature of FAS could be searched for in NS-FASD to improve the specificity of the diagnosis.

## Data availability statement

The data analyzed in this study is subject to the following licenses/restrictions: The datasets presented in this article are not readily available because the data that support the findings of this study are available on request from the corresponding author. The data are not publicly available due to privacy or ethical restrictions. Requests to access these datasets should be directed to JF, justine.fraize@inserm.fr or to DG, david.germanaud@cea.fr.

## Ethics statement

The studies involving humans were approved by Paris-Saclay research ethics committee (CER-Paris-Saclay-2020-094). Controls’ data were used within the framework of the ethical authorizations of the primary studies (Gene and autism, Inserm C07-33). The studies were conducted in accordance with the local legislation and institutional requirements. The ethics committee/institutional review board waived the requirement of written informed consent for participation from the participants or the participants’ legal guardians/next of kin because written informed consent from the FASD participants’ legal guardian/next of kin was not required to participate in this study in accordance with the national legislation and the institutional requirements. Written informed consent from the control participants’ legal guardian/next of kin was required to participate in the initial study in accordance with the national legislation and the institutional requirements.

## Author contributions

JF: Conceptualization, Formal analysis, Investigation, Methodology, Resources, Software, Validation, Visualization, Writing – original draft, Writing – review & editing. YL: Methodology, Supervision, Validation, Visualization, Writing – review & editing. ME-B: Data curation, Resources, Writing – review & editing. EK: Validation, Writing – review & editing. RD: Resources, Writing – review & editing. LH-P: Conceptualization, Supervision, Validation, Writing – review & editing, Funding acquisition. JL: Conceptualization, Methodology, Software, Supervision, Validation, Visualization, Writing – review & editing. DG: Conceptualization, Data curation, Funding acquisition, Methodology, Resources, Supervision, Validation, Writing – review & editing.

## Footnotes

^1^https://brainvisa.info/
