# Supplementary material for: Spectral-based thickness profiling of the corpus callosum enhances anomaly detection in fetal alcohol spectrum disorders
Source: Front Neurosci. 2023 Nov 6;17:1289013. doi: 10.3389/fnins.2023.1289013 (PMC10657855; doi:10.3389/fnins.2023.1289013)
Supplement: Supplementary file 1 [file Data_Sheet_1.docx]

Spectral-based thickness profiling of the corpus callosum enhances anomaly detection in fetal alcohol spectrum disorders

*Spectral-based thickness profiling of the corpus callosum in fetal alcohol spectrum disorders*

Justine Fraize^1,2*^, Yann Leprince^1^, Monique Elmaleh-Bergès^3^, Eliot Kerdreux^1,2^, Richard Delorme^4^, Lucie Hertz-Pannier^1,2^, Julien Lefèvre^†5^, David Germanaud^†1,2,6*^

^1^ CEA Paris-Saclay, Frederic Joliot Institute, NeuroSpin, UNIACT, Centre d'études de Saclay, Gif-sur-Yvette, France

^2^ Université Paris Cité, Inserm, NeuroDiderot, InDEV, Paris, France

^3^ Department of Pediatric Radiology, Centre of Excellence InovAND, AP-HP, Robert-Debré Hospital, Paris, France

^4^ Department of Child and Adolescent Psychiatry, Centre of Excellence InovAND, AP-HP, Robert-Debré Hospital, Paris, France

^5^ Aix-Marseille Université, CNRS, Institut de Neurosciences de La Timone, UMR7289, Marseille, France

^6^ Centre de Référence Déficiences Intellectuelles de Causes Rares, Department of Genetics, Centre of Excellence InovAND, Robert-Debré Hospital, AP-HP, Paris, France

^†^These authors have contributed equally to this work

# Supplementary material


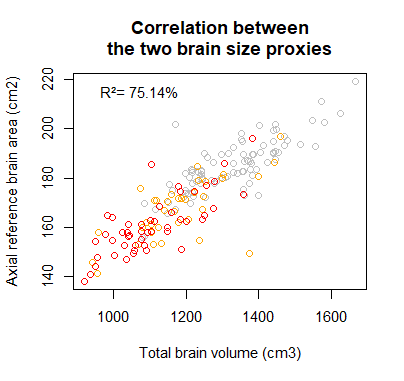


**Figure 1.** Correlation between the two proxies of brain size, the total brain volume from Volbrain and the axial reference brain area (measurement previously described in Fraize et al., 2023c. Top left, R² coefficient of correlation. Controls in grey, FAS in red, NS-FASD in orange.


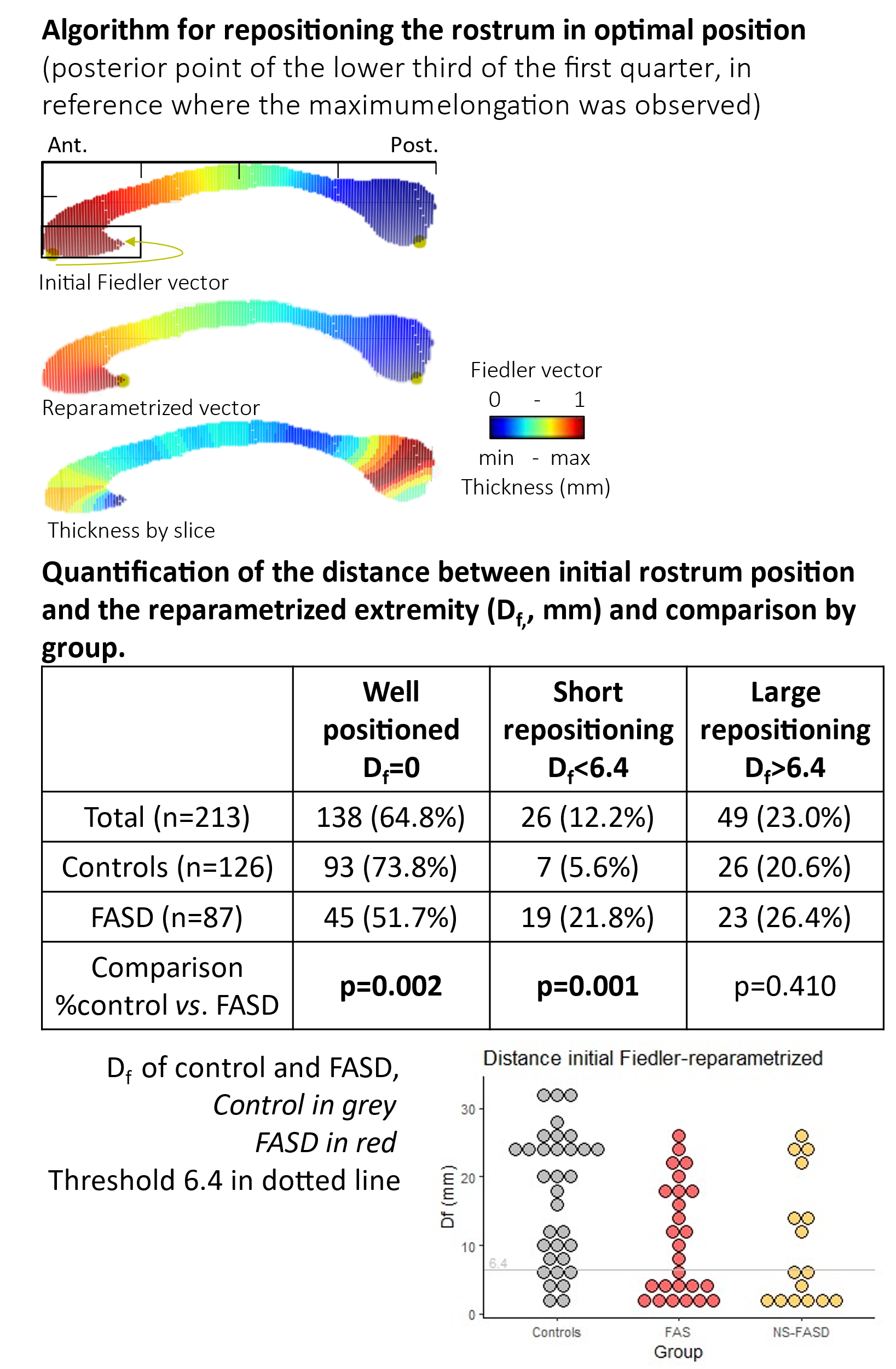


**Figure 2.** Algorithm for repositioning the rostrum in optimal position.

Table 1. Manual correction of the mask of the corpus callosum by group.

|  | **FAS**  n = 52 | **NS-FASD**  n = 37 | **Controls**  n = 126 |
| --- | --- | --- | --- |
| Manually corrected | 30.8% | 32.4% | 62.7% |

Table 2. Effect of scanner, age, and sex on raw measured parameters in the control group.

|  | **Age effect**  **p^a^** | **Sex effect**  **p^a^** | **Scanner effect**  **p^a^** |
| --- | --- | --- | --- |
| Length of the corpus callosum | **0.009** | **0.043** | **0.033** |
| Genu thickness | 0.659 | 0.186 | **0.022** |
| Body hump thickness | 0.659 | **0.015** | **0.021** |
| Body median thickness | 0.659 | **0.010** | **0.002** |
| Isthmus thickness | 0.058 | **0.001** | **0.001** |
| Splenium thickness | **0.001** | 0.036 | 0.450 |

Multivariate linear regression.

Bold type, p < 0.05, after FDR correction.

**^a^** *p*-value from the generalized linear model including scanner, age, and sex as covariates for each parameter measured.

Table 3. Percentage change due to site effects correction per Combat.

|  | Correction (%) |
| --- | --- |
| Length of the corpus callosum | -0.09 |
| Genu thickness | 0.31 |
| Body hump thickness | -3.06 |
| Body median thickness | 0.45 |
| Isthmus thickness | 0.71 |
| Splenium thickness | 0.07 |

Correction = mean of (corrected value - native value)/ native value, expressed as a percentage.
